# Longitudinal assessment of cardiac function in infants with Down’s syndrome using novel echocardiography techniques – project protocol

**DOI:** 10.12688/hrbopenres.13168.1

**Published:** 2020-10-21

**Authors:** Aisling Smith, Eleanor Molloy, Jan Miletin, Anna Curley, Joanne Balfe, Orla Franklin, Afif EL-Khuffash

**Affiliations:** 1 Department of Neonatology, The Rotunda Hospital, Dublin, Dublin, D01P5W9, Ireland; 2 Department of Paediatrics and Child Health, Trinity College Dublin, Dublin, Dublin, D02PN40, Ireland; 3 Department of Neonatology, Coombe Women and Infants University Hospital, Dublin, Dublin, D08XW7X, Ireland; 4 Department of Neonatology, The National Maternity Hospital, Dublin, Dublin, D02YH21, Ireland; 5 Department of Paediatrics, Tallaght University Hospital, Dublin, Dublin, D24NR0A, Ireland; 6 Department of Paediatric Cardiology, Our Lady’s Children’s Hospital, Crumlin, Dublin, Dublin, D12N512, Ireland; 7 School of Medicine, Royal College of Surgeons in Ireland, Dublin, Dublin, D02P796, Ireland

**Keywords:** Down Syndrome, Trisomy 21, Pulmonary arterial hypertension, Myocardial Function

## Abstract

**Background:** Down’s syndrome (DS) is the most common chromosomal abnormality globally. Ireland has one of the highest rates of DS in the western world with an incidence of 1:444 live births. Congenital heart disease (CHD) and pulmonary hypertension (PH) are the commonest morbidities affecting the cardiovascular system in DS. PH is associated with significant morbidity and an increase risk of mortality. The impact of the diagnosis of DS, the presence of CHD and the associated PH on myocardial function during transition and over the first 2 years of age in this population is not well defined and warrants further study. In particular, serial measurements of pulmonary pressures in this population over the first week of age are lacking. This study aims to characterise myocardial function and pulmonary haemodynamics in infants with Down syndrome during the transitional period (over the first week of age) and throughout the first two years of age.

**Methods: **A prospective, observational study utilising novel echocardiography techniques to assess myocardial function and pulmonary haemodynamics over the first two years of age in infants with Down Syndrome. A population of healthy infants without CHD or a diagnosis of DS will be recruited as controls. This study will be conducted across the three Dublin maternity units.

**Discussion: **In total, 70 babies with DS have been enrolled into this study with 292 echocardiograms performed to date. Further evaluation of cardiac performance in DS infants with and without CHD may yield more insight into the pathophysiology of cardiac dysfunction and pulmonary hypertension that are recognised features in these patients. This could aid in our ability to monitor and treat patients, as well as improve our ability to predict outcomes.

## Introduction

Down’s syndrome (DS) is the most common chromosomal abnormality globally. The incidence of DS in Ireland is 1:444 live births, the highest in Europe ^1^. One of the major morbidities associated with this diagnosis is congenital heart disease (CHD) and pulmonary hypertension (PH). Approximately 50% of these children will have CHD, the most frequent of which is an atrioventricular septal defect (AVSD, 50%), followed by ventricular septal defect (VSD, 30%) ^2, 3^. PH complicates a significant number of infants with CHD, adding an additional layer of complexity to their management.

The development of later onset PH in this population is well recognised. However, there is increasing evidence that infants with DS without CHD (DS-No CHD) have a 10-fold increased risk of developing neonatal PH with an incidence of up to 5% ^3– 5^. The aetiology of early PH in infant with DS is multifactorial: failure of vascular remodelling of pulmonary vessels following birth, the persistence of fetal capillary networks and a reduction of the cross sectional area of the vascular bed coupled with a decreased alveolar density results in the persistence of the high pulmonary vascular resistance (PVR) and pulmonary arterial hypertension ^6^. In addition, impaired vascular endothelial function results in a reduction in endogenous nitric oxide (eNO) production and a reduced sensitivity to eNO ^7^. Recurrent respiratory tract infections, chronic obstructive airway disease and the persistence of intra- and extra-cardiac left to right shunts can lead to chronic pulmonary hypertension. This is coupled with a thinner tunica media layer present the pulmonary vascular which makes this population more susceptible to sheer stress damage caused by increased pulmonary blood flow ^8^.

Pulmonary hypertension in infants with Down Syndrome is associated with an increase in respiratory morbidity and an increased mortality. This manifests as a higher risk of requiring extra-corporeal membrane oxygenation (ECMO) due to respiratory failure following respiratory tract infections than in the general infant population. The risk of death is also higher in this group following ECMO (35% vs. 25%) ^9^. In addition, the mortality rate of critically ill infants and children with DS admitted to intensive care is high (40%) with respiratory morbidity associated with PH being the leading cause ^10^. The progression of PH during the early neonatal period and over the first two years of age is poorly characterised in this population. The true incidence of PH in infants with DS with and without CHD is likely to be underestimated.

The impact of the diagnosis of DS, the presence of CHD and the associated PH on myocardial function during transition and over the first 2 years of age in this population is not well characterised. In particular, serial measurements of pulmonary pressures in this population over the first week of age are lacking. There is evidence for the presence of global (systolic and diastolic) left and right ventricular dysfunction in DS during fetal life irrespective of the presence of CHD ^11^. Examination of DS fetal hearts (without CHD) reveals a shorter ventricular septum, a larger membranous septum and dysplasia of the atrioventricular valves which may explain the reduced function identified during fetal life ^12^. Myocardial dysfunction is also identified in infants with DS beyond the second year of age. Recent studies demonstrated reduced left ventricle (LV) diastolic function and reduced right ventricle (RV) systolic and diastolic function identified using conventional Doppler methods and tissue Doppler Imaging (TDI) in DS-No CHD infants at around 10 years of age ^13, 14^. However, there is a distinct lack of literature describing the change in myocardial performance in DS infants (with and without CHD) during the critical transitional period and over the first two years of age. In addition, the impact of PH on LV and RV function, and their relationship with important outcomes required further study.

Monitoring the cardiovascular status of infants with DS remains a challenge due to the insensitivity of clinical indicators in defining systemic perfusion, and identifying the presence of CHD, PH and myocardial dysfunction ^15– 17^. There are also limitations of conventional echocardiography functional parameters, such as shortening fraction (SF) and ejection fraction (EF) in assessing myocardial performance ^18^. In this study we will use novel methods of functional assessment to better characterise myocardial function in infants with Down syndrome during the transitional period (over the first week of age) and throughout the first two years of age. The newer methods of functional assessment will include: tissue Doppler imaging (TDI) and tissue Doppler-derived deformation analysis; speckle tracking echocardiography (STE) to measure deformation and left ventricular (LV) rotational mechanics; right ventricle (RV) specific parameters including fractional area change (FAC) and tricuspid annular plane systolic excursion (TAPSE). Those newer methods of myocardial assessment are more sensitive in detecting myocardial dysfunction in various disease states in the neonatal population and in children and can facilitate the early detection of subclinical myocardial dysfunction before the appearance of clinically significant disease ^19, 20^. In addition to the functional assessment, estimation of pulmonary arterial pressure using echocardiography is now feasible. Pulmonary artery acceleration time (PAAT) has recently been validated in children against right heart catheter (RHC) techniques. There is a significant inverse relationship between PAAT and RHC-derived measures of pulmonary arterial pressure ^21^. This provides a unique opportunity to accurately characterise longitudinal changes in pulmonary arterial pressure in this population. Further evaluation of cardiac performance in DS infants with and without CHD may yield more insight into the pathophysiology of cardiac dysfunction and pulmonary hypertension that are recognised features in these patients. This could aid in our ability to monitor and treat patients, as well as improve our ability to predict outcomes.

The aim of this prospective, observational study is to serially assess LV and RV function in infants with Down syndrome (with and without CHD) using tissue Doppler imaging, deformation imaging and RV-specific functional parameters over the first two years of age and to track the change in pulmonary arterial pressure in this population over the same time period, and compare those measurements to a cohort of healthy controls. We also aim to assess the ability of those echocardiography measurements to predict important outcomes including respiratory morbidity, clinically evident PH needing medical therapy, hospital admissions, need for ECMO, neurodevelopmental outcome at two years, and death.

## Research design and methodological approach

### Study design

This study will be a prospective observational cohort study carried out in the Dublin tertiary neonatal intensive care units of the Rotunda Hospital, The National Maternity Hospital and the Coombe Women’s Hospital. The three maternity hospitals deliver approximately 80 infants with a diagnosis of DS per year. The majority of infant with DS are identified antenatally through the routine antenatal scans. Recruitment and the initial echocardiography scans (3 over the first week of age) will be carried out in the maternity hospital of recruitment. The subsequent echocardiography scans (a further 3 over the first two years of age) will be a carried out at the Rotunda Hospital, Dublin.

### Study population

 All infants with a confirmed diagnosis of DS made antenatally, or when a clinical diagnosis of DS made postnatally will be included in this study. In addition, a cohort of healthy term infants will be recruited from the Rotunda Hospital to serve as a control population. Healthy controls are infants without a diagnosis of DS (and born > 37 weeks gestation) born to mothers without significant maternal illness (diabetes of any type, pre-eclampsia, hypertension, clinical chorioamnionitis, or absent/reversed end diastolic flow in the umbilical arteries anytime during the pregnancy). Infants will be excluded if there is a lack of parental consent, or if there is a high likelihood of death over the first week of age. The healthy term cohort will undergo the echocardiography assessments at the six time points outlined below in addition to the developmental assessments at 6, 12 and 24 months of age.

### Data collection and outcome assessments

Following recruitment, important antenatal, birth and neonatal characteristics will be collected. This will occur in the local maternity hospital ( Table 1). Infants will be followed up at 6 and 12 months of age to undergo an echocardiogram and neurodevelopmental assessment at 12 months of age ( Table 2). Infants will undergo a final assessment at 24 months of with an echocardiogram ( Table 3).

**Table 1.** Collection of clinical demographics and outcomes: first week of age.

Data	Output		
Maternal Age	Years		
Parity	Number		
Gender	Male/Female		
Fetuses	Singleton/Twins/Triplets		
Mode of Delivery	Vaginal/Caesarean Section		
1 and 5 Minute Apgar score	1 – 10		
Cord pH	Number		
Surfactant administration	Yes/No		
Need for Cardiopulmonary resuscitation	Yes/No		
Need for adrenaline	Yes/No		
Chorioamnionitis	Yes/No		
Pre-eclampsia	Yes/No		
Absent end diastolic flow	Yes/No		
Polyhydramnios	Yes/No		
Oligohydramnios	Yes/No		
Antepartum Haemorrhage	Yes/No		
Prolonged Rupture of Membranes	Yes/No		
Antenatal steroids	None/one dose/two doses		
Antenatal Diagnosis of DS	Yes/No		
Atrioventricular septal defect	Yes/No		
Ventricular septal defect	Yes/No		
Atrial septal defect	Yes/No		
Other Cardiac Lesion	Narrate		
None Cardiac Lesions	Narrate		
Sepsis	Yes/No		
Inotropes	Yes/No		
Nitric Oxide	Yes/No		
Pulmonary Hypertension	Yes/No		
Intubation	Yes/No		
Mode of Ventilation	High Frequency/Conventional		
Ventilation Days	Number		
Continuous Positive Airway Pressure Days	Number		
Oxygen Days	Number		
Total Parenteral Nutrition Days	Number		
Time to full feeds (Days)	Number		
Hospital Days	Number		
ECMO referral	Yes/No		
First & Discharge Blood Gas	pH/pCO _2_/pO _2_/HCO _3_/Base Excess		
First & discharge Full Blood Count	Haemoglobin/Platelets/Whit Cells/Neutrophils		
First & discharge Renal/Liver Profile	Na/K/Urea/Creatinine/Bilirubin/AST/ALT		
First and Discharge oxygen saturations	Number		
Discharge Medications	Narrate		
Echocardiography	Day 1	Day 2	Day 5 – 7

**Table 2.** Assessment at 6 months and 12 months of age.

Data	Output
Eltroxin	Yes/No
Number of Hospital Admissions	Number
Reason for Hospital Admissions	Narrate
Heart Defect (if present) corrected	Yes/No
Duration of cardiac intensive care Stay	Days
Current medications	Narrate
Current Oxygen Saturations	Number
Developmental Examination	6 months and 12 months
Echocardiogram	6 months and 12 months
Neurodevelopmental Assessment	

**Table 3.** Final assessment at 24 months of age.

Data	Output
Eltroxin	Yes/No
Number of Hospital Admissions	Number
Reason for Hospital Admissions	Narrate
Heart Defect (if present) corrected	Yes/No
Duration of cardiac intensive care Stay	Days
Current medications	Narrate
Current Oxygen Saturations	Number
Echocardiogram	24 months

### Echocardiography assessment

The echocardiography scans will be performed at six time periods. Three times over the first week of age: Day 1 [12 – 24 hours], Day 2 [36 – 48 hours] and Day 5 – 7; and three times after the first week of age: 6 months (± 1month); 12 months (± 1month); and 24 months (± 1month). Evaluations will be performed using the Vivid echocardiography system (GE Medical, Milwaukee) and a cardiology multi-frequency probe. The echocardiography machine and the necessary probes, in addition to the expertise necessary for image acquisition and analysis are already available. The echo machine is primarily dedicated to research and will therefore be available for the study. No sedation will be used. Image acquisition will only require 10 minutes of scanning. If the infant is admitted to the neonatal intensive care unit, the echocardiography study will be performed there. All scans will be recorded on the machine’s internal hard drive and transferred to the Echo PAC archiving system for offline measurements and validation.

Analysis of the parameters will be done while the assessor is blinded to the clinical condition of the infant. The first echocardiogram of each infant will undergo a formal examination to exclude any structural heart defects. Studies will be performed using standard neonatal windows including apical, parasternal, subcostal, and high parasternal windows. All scans will be performed by trained personnel. 

Our group and collaborators recently demonstrated the feasibility, reliability and validity of tissue Doppler Imaging (TDI) and strain using speckle tracking echocardiography (STE), in addition to rotational mechanics in the term and preterm population. In addition, we detailed the techniques used to obtain the images, offline analysis and measurements ^22, 23^.

### Developmental assessment

All infants with have standardised clinical developmental follow up at 6, 12, and 24 months of age followed by an assessment by the paediatric developmental psychologist. Details of developmental follow-up and early intervention will be collated. The Bayley-III cognitive, language, and motor composite scores (CSs) will be completed by a dedicated trained psychologist. The administration of the test will follow the Bayley Manual guidelines. The Bayley Scales are standardized tests and demonstrate excellent inter-observer reproducibility. The Bayley III examines the following developmental subsets: cognitive, receptive communication, expressive communication, fine motor and gross motor attributes ^24^.

### Outcome measures

Our primary outcome is the development of LV and RV dysfunction in addition to pulmonary hypertension over the study period. Pulmonary hypertension will be defined as follows: Normal Structural anatomy of the heart on echocardiogram; and requirement of at least 0.4 fractional inspired oxygen to maintain a preductal saturation of ≥ 95%; or, in the presence of tricuspid regurgitant (TR) jet, an estimated right ventricular systolic pressure (using the Bernoulli Equation) ≥ 50% of the systemic systolic pressure measured at the start of the echocardiogram; or in the presence of a patent ductus arteriosus (PDA), a demonstration of bidirectional flow across the vessel or right to left flow; or in the absence of a TR jet or a PDA, an intraventricular septum bowing into the left ventricular cavity or a pulmonary artery acceleration time < 40ms. Myocardial dysfunction identified on any of the measurements described above will be diagnosed if the obtained value falls below 2 standard deviations of the mean reference value according to published literature ^25^.

 In addition, we will assess the association between pulmonary hypertension, myocardial dysfunction and important clinical outcomes occurring throughout the study period:

- Clinical pulmonary hypertension requiring medication
- Respiratory morbidity and associated hospital admissions
- Admission to a paediatric intensive care unit
- Need for ECMO
- Adverse neurodevelopmental outcome identified on Bayley’s assessment
- Death within the first two years of age

### Definition end of study

 The end of the study will be the date of the last visit of the last subject anticipated to be 2 years after enrolment of the first subject. The study sponsors and/or the study steering committee have the right at any time to terminate the study for clinical or administrative reasons. The end of the study will be reported to the Research Ethics Committee (REC) within 90 days, or 15 days if the study is terminated prematurely. The investigators will inform subjects and ensure that the appropriate follow-up is arranged for all involved. A summary report of the study will be provided to the REC within 6 months

### Sample size

 Currently, the incidence of pulmonary hypertension in infants with DS is about 5%. However, this may be a significant under estimation. The incidence of myocardial dysfunction in infants with DS during the early neonatal period and into the second year of age is currently unknown. Therefore, no power calculations are possible in this cohort and as a result, the sample size of the cohort will be determined by the incidence of Down syndrome in the three Dublin maternity hospitals. DS has an incidence of 1:444 live births in Ireland which is the highest in Europe ^1^. The three Dublin maternity hospitals will deliver about 80 infants with DS annually. As a result, we aim to recruit 120 infants with DS over a two year period (50% recruitment rate). The incidence of congenital heart disease in the DS population is 45 – 50% ^2, 3^. As a result, we anticipate having 60 infants with DS and CHD and 60 infants with DS but without CHD. In addition, we aim to recruit 60 healthy term controls matched for gestation and birthweight to infants with DS without CHD over the same time point. This provides a total study sample size of 180 infants.

### Statistical analysis

All the analysis will be conducted by a biostatistician with expertise in large scale observational studies. The cohort will be divided into three groups: Infants with DS and CHD (DS-CHD Group); infant with DS without CHD (DS no CHD Group) and healthy controls (Control Group). Continuous data will be tested for normality using the Shapiro-Wilk test and a histogram representation of data and summarised as means (standard deviation) or medians [inter-quartile range] as appropriate. Categorical data will be summarised as counts (%). Two group analyses will be conducted using the Student t-test, the Mann Whitney U test as appropriate, or Chi square test as appropriate. Three group analyses will be conducted using one way ANOVA or the Kruskal-Wallis Test. Serial data will be compared using two way ANOVA with repeated measures. Regression analysis will be conducted to assess the independent effect of predictor variables on important outcomes. A receiver operating characteristic (ROC) curve will be constructed to assess the ability of the various echocardiography parameters to predict important outcomes. We will use SPSS version 23 to conduct the analyses. Statistical significance is achieved with a p value < 0.05.

### Dissemination and knowledge exchange plan

The output of this project can have a significant impact on the monitoring of infants with DS over the first two weeks of age and as such, a robust plan for dissemination and knowledge exchange will be developed and include:

- Parent information Sessions and focus groups with multidisciplinary staff. The key stakeholders of this process (parents and their infants, healthcare professionals, allied health staff, nursing) will be invited to a symposium which will be set up to present the findings to all relevant stakeholders and the wider medical community. Sessions will be geared to cater for particular interest groups (e.g patients/parents vs. nursing, vs. medical).
- Presentation at national and international paediatric and neonatal meetings. We will target the largest international meetings in both in North America and Europe. In North America, we will submit the results of the study to be presented at the Paediatric Academic Societies (PAS) meeting which an annual event attracting over 8,000 delegates. The principal investigator (PI) of this grant application is a member of the steering committee of the Haemodynamic Section of PAS and can have a say in what is presented at those events. In addition, we will submit the data to the newly relabelled European Society of Paediatric Research (ESPR) soon to be known as the European Board of Neonatology. The principal investigator of this grant application sits on the Circulation Section of ESPR. This provides the opportunity to organise focussed symposia on certain topics for the purposes of knowledge dissemination.
- Publications in peer-reviewed journals and the medical press using both basic science and translational journals. We aim to publish the findings of the study in an easy to understand format. We also plan to compose a review article on the topic where we suggest a guideline for the close monitoring of the cardiovascular status of infants with DS.
- Neonatal Echocardiography Skills Work Shops and Study Days: Three of the co-applicants on this grant in addition to the PI have successfully coordinated echocardiography workshops. We will devise a workshop specific to the skills needed to perform this comprehensive echocardiography assessment to facilitate the rolling out of the monitoring program.
- Web-based learning: We will create a dedicated website to contain patient and parent information leaflets, data and results of the study (both in medical and lay terms), information on access to future dedicated follow up clinics.

## Ethics

Ethical approval has been sought and granted to perform this study in the Rotunda Hospital (REC-2016-006), The Coombe Hospital (Study No 19 – 2017) and The National Maternity Hospital Holles St. (EC 21.2017). Data will be entered numerically or as a dichotomous variable. The subjects will be identified by a study specific subject number in the database. The name and any other identifying detail will not be included in any study data electronic file. No individual patient data will be presented. Written parental consent will be sought and confirmed for each study participant.

## Study status

 To date ethical approval has been sought and granted to perform this study in the Rotunda Hospital, The Coombe Hospital and The National Maternity Hospital Holles St. The study has received financial support from a Health Research Board (HRB) grant and Crumlin Research Education Support Grant. Education sessions have been held across the three study sites to inform staff of the study and to encourage recruitment of all babies born with DS in Dublin over the study period. Recruitment began in The Rotunda Hospital July 2018 and in The Coombe Hospital and National Maternity Hospital, Holles St began in January 2019.

In total, 70 infants with Down Syndrome have enrolled into the study. 292 echocardiography studies have been performed. Of the 292 echocardiograms performed, 58 were obtained on Day 1, 67 on Day 2 and 66 on Day 3 post delivery. There have been 45 six month echocardiograms, 41 one year echocardiograms and 15 two year echocardiograms carried out to date. Offline analysis of the echocardiograms to evaluate myocardial function and pulmonary haemodynamics has begun.

We have enrolled 60 healthy control infants into the study with 268 control echocardiography studies performed. Of the 268 echocardiography studies performed 60 were obtained on Day 1, 58 on Day 2 and 52 on Day 3 post delivery. A further 50 6 month scans and 44 one year control echocardiograms and 4 two year control echocardiograms have been performed to date. Offline analysis of control echocardiograms has also commenced.

## Discussion

There is a distinct lack of information on myocardial performance during the transitional period and over the first two years of age in infants with DS with and without CHD. In addition, the longitudinal changes in pulmonary arterial pressure and pulmonary vascular resistance in this population is not well documented. In Ireland, we have a unique opportunity to describe myocardial performance and pulmonary haemodynamics in infants with DS. As described above, we have the highest incidence of DS in Europe with a significant proportion born in the three Dublin Maternity Hospitals. In addition, our groups and collaborators have pioneered the use of novel echocardiography markers in the assessment of left and right ventricle function in addition to pulmonary artery haemodynamics. This project falls in line with the objectives of the National Children’s Hospital Foundation. The project will be significantly relevant to a vulnerable group of patients of the Hospital, their parents and those involved in the delivery of their care. Currently, infants with DS without CHD do not undergo regular echocardiography beyond the neonatal period, and potential myocardial dysfunction/elevated pulmonary pressures may go undiagnosed. In this cohort, pulmonary hypertension and myocardial dysfunction can present overtly at an advanced stage in association with respiratory tract infections over the first two years of age. Infants with DS and CHD are at a continued risk of developing pulmonary hypertension and myocardial dysfunction following surgical repair. Regular follow up of this population would also yield the same benefits.

Characterising those important haemodynamic changes in DS infants with and without CHD, and relating those finding to important clinical outcomes can pave the way for earlier prophylactic therapeutic interventions and reduce the risk of the development of significant morbidity, in particular respiratory morbidity, need for intensive care admission, and need for ECMO. This will have significant benefits for those infants and their families. Optimising the medical care of infant with DS thought out the first two years of age can have a substantial impact on their neurodevelopmental outcomes, as they will spend less time in hospital and more time receiving important interventions. In addition the reduction in morbidity outlined above can lead to a significant cost saving for the health services in this country where the extra funds generated from the reduced hospital admissions can be redirected to optimising early intervention services for those infants. 

Upon the completion of this project, the data generated can provide the necessary stepping stone for a dedicated cardiovascular monitoring and intervention program for those infants. This new healthcare program will be innovative and has the potential to influence government policy and highlight the need for further investment into the care of those infants. The program will be based in the National Children’s Hospital and integrated into a comprehensive follow up program for infants with DS, leading to an improvement of healthcare delivery for Irish Children with DS. The collaborative nature of this project, which involves personnel from the three Dublin maternity Hospitals (The Rotunda Hospital; The National Maternity Hospital; and The Coombe Women and Infants Hospital), and Our Lady’s Children’s Hospital, Crumlin, is a perfect example of how healthcare professionals caring for a vulnerable group of infants can pool expertise and resources to improve outcomes.

## Data availability

### Underlying data

No data are associated with this article.
