# Supplementary material for: Longitudinal assessment of cardiac function in infants with Down’s syndrome using novel echocardiography techniques – project protocol
Source: HRB Open Res. 2020 Oct 21;3:77. [Version 1] doi: 10.12688/hrbopenres.13168.1 (PMC8145226; doi:10.12688/hrbopenres.13168.1)
Supplement: Peer review history

# Peer review: Reviewer response for version 1

**DOI:** 10.21956/hrbopenres.14292.r28197

**Published:** 2021-05-24

**Authors:** Neil Patel

**Affiliations:** 1 Department of Neonatology, Royal Hospital for Children, Glasgow, UK

This prospective cohort-control study is investigating the natural history of pulmonary hypertension and cardiac function in infants and children with Down Syndrome over the first year of life.

 This is an important area of investigation, which may lead to improved management and outcomes in Down Syndrome, particularly in the first years of life.

 The investigating team are uniquely-placed to conduct this study due to their expertise in functional haemodynamic assessment and the high incidence of Down Syndrome in Ireland and managed at their institutions.

 The study is already progressing well with very good recruitment of case and control groups and collection of longitudinal data as planned in the original protocol.

 I have a few specific observations and comments:

 Sample size: The sample size is based on the number of suitable participants recruited during the study period, rather than a predicted difference in primary outcome measure. Whilst this is an understandable, practical approach it raises the possibility that any observed differences in outcomes between cases and controls may not reach statistical significance.

 Potential modifying factors:

 Pre and postnatal variables may also be important determinants of PH and cardiac function. For example maternal factors (e.g maternal disease, medications including SSRI) and postnatal factors including nutrition, gastro-oespageal reflux, oxygen therapy, pulmonary vasodilator therapy (e.g. sildenafil).

 Neurodevelopmental assessment:

 Will the standard Bayley-III be used for Down Syndrome and Control groups? Have the authors considered use of a modified scale for infants with Down Syndrome? (Moore *et al*). ^1^

Is the study design appropriate for the research question?

Yes

Is the rationale for, and objectives of, the study clearly described?

Yes

Are sufficient details of the methods provided to allow replication by others?

Yes

Are the datasets clearly presented in a useable and accessible format?

Yes

Reviewer Expertise:

Neonatal haemodynamics including cardiac function and pulmonary hypertension

I confirm that I have read this submission and believe that I have an appropriate level of expertise to confirm that it is of an acceptable scientific standard.

## References

[1] : A modified version of the Bayley Scales of Infant Development-II for cognitive matching of infants with and without Down syndrome. *J Intellect Disabil Res*.2008;52(Pt 6) : 10.1111/j.1365-2788.2008.01064.x 554-61 10.1111/j.1365-2788.2008.01064.x 18444985

# Peer review: Reviewer response for version 1

**DOI:** 10.21956/hrbopenres.14292.r28195

**Published:** 2020-10-23

**Authors:** Koert de Waal

**Affiliations:** 1 Department of Neonatology, University of Newcastle, Newcastle, Australia

The authors describe a study protocol with aim to describe transitional hemodynamics and post-discharge hemodynamics in follow up of infants with Down syndrome. The protocol provides the rationale for the study, especially the longer term follow up and the association with PH, and has all details as would be required to fulfil the aims of the study.

 Recruitment is well underway, and publications from this team are already available using this protocol.

 I have no major comments, some questions: 

 Could the authors expand on the rationale for the 3 scans early after birth? Especially for infants who do not need any respiratory support after birth, this frequency seems a bit much as one scan on day 5 would be enough to determine the baseline HD status and estimated pulm pressures.

 According to the consensus definition, PH can be diagnosed from 3 months of age. Yet, the authors choose 6 months as first measure, was this intentional?

Is the study design appropriate for the research question?

Yes

Is the rationale for, and objectives of, the study clearly described?

Yes

Are sufficient details of the methods provided to allow replication by others?

Yes

Are the datasets clearly presented in a useable and accessible format?

Yes

Reviewer Expertise:

Neonatology, neonatal hemodynamics, neonatal cardiac ultrasound.

I confirm that I have read this submission and believe that I have an appropriate level of expertise to confirm that it is of an acceptable scientific standard.

# Response

**Published:** 2020-10-23

**Authors:** Aisling Smith

**Affiliations:** The Rotunda Hospital, Ireland

Dear Prof. De Waal,

Many thanks for your comments. We performed three scans over the first week of age as serial data assessing pulmonary haemodynamics and myocardial function over the early newborn period in this cohort is currently lacking. In addition, we were keen to track such changes and assess if pulmonary vascular resistance fell at all between the first to third scan. 

Regarding inviting patients back at 6 months of age this was done for practical reasons. Infants with T21 often have many clinic appointments and are getting established with multidisciplinary teams in their early months of age. We did not want the research scan to be an extra request on parents during these busy months so decided to invite them back at 6 months of age. 

Best wishes,

Aisling

# Response

**Published:** 2021-01-26

**Authors:** Aisling Smith

**Affiliations:** The Rotunda Hospital, Ireland

In response to the first query, we wanted to serially assess transitional changes occurring shortly after birth as many infants with T21 still have evidence of pulmonary hypertension by day 5 based on our preliminary data and we required an equal number of echocardiograms on the control infants in order have have directly comparable data sets. 

 In response to the second query, deferring the 4th echocardiogram to 6 months was intentional as many of our infants with T21 are undergoing assessments at 3 months of age with the Irish cardiac centre in preparation for surgical correction of congenial heart defects and we didn't want to place an extra burden of assessments on families at this time.
